# Significance of Circular FAT1 as a Prognostic Factor and Tumor Suppressor for Esophageal Squamous Cell Carcinoma

**DOI:** 10.1245/s10434-021-10089-9

**Published:** 2021-06-29

**Authors:** Wataru Takaki, Hirotaka Konishi, Katsutoshi Shoda, Tomohiro Arita, Satoshi Kataoka, Jun Shibamoto, Hirotaka Furuke, Kazuya Takabatake, Hiroki Shimizu, Shuhei Komatsu, Atsushi Shiozaki, Hitoshi Fujiwara, Kiyoshi Masuda, Eigo Otsuji

**Affiliations:** 1 Division of Digestive Surgery, Department of Surgery, Kyoto Prefectural University of Medicine, Kyoto City, Japan; 2 First Department of Surgery, Faculty of Medicine, University of Yamanashi, Chuo, Yamanashi Japan; 3 Kawasaki Medical School, Kurashiki City, Okayama Japan

## Abstract

**Background:**

Circular RNA is a novel endogenous non-coding RNA with a stable loop structure, and theories for its biogenesis and usefulness as a biomarker in various cancers have been proposed. The present study investigated the significance of circular FAT1 (circFAT1) as a novel biomarker in esophageal squamous cell carcinoma (ESCC).

**Method:**

CircFAT1 expression levels were measured in ESCC cell lines and the effects of downregulating circFAT1 on cell migration and invasion were examined using a transwell assay. The functions of miR-548g, which will be sponged by circFAT1, were assessed. Furthermore, the expression of circFAT1 was evaluated in 51 radically resected ESCC tissue samples using quantitative reverse transcription polymerase chain reaction (qRT-PCR). The relationships between circFAT1 expression, clinicopathological factors, and patient prognosis were analyzed.

**Results:**

CircFAT1 expression levels were significantly lower in tumor tissue than in adjacent non-tumorous mucosal tissue (*p* = 0.01). The downregulation of circFAT1 expression promoted ESCC cell migration and invasive ability, but not proliferation. The expression of miR-548g was upregulated by the downregulation of circFAT1. The overexpression of miR-548g also promoted ESCC cell migration and invasion. Recurrence-free survival (*p* = 0.02) and cancer-specific survival (*p* = 0.04) rates were significantly higher in patients with elevated circFAT1 expression levels.

**Conclusion:**

The expression level of circFAT1 is a novel prognostic marker in ESCC patients. New treatment strategies may be developed using the tumor suppressive functions of circFAT1.

**Supplementary Information:**

The online version contains supplementary material available at 10.1245/s10434-021-10089-9.

Esophageal cancer, a high-grade malignancy, is the sixth leading cause of cancer-related death worldwide.^1^ Squamous cell carcinoma is the dominant histological type, particularly in Asia, and its incidence is increasing. Despite advances in multidisciplinary treatments, recurrence is common among patients with esophageal squamous cell carcinoma (ESCC) and thus their prognosis is poor.^2^ Further studies are needed to elucidate the biological and molecular mechanisms underlying ESCC in order to identify new diagnostic and prognostic biomarkers and develop novel therapeutic targets.

Circular RNA (circRNA) is a non-coding RNA with a closed loop structure that lacks a 5′ cap and 3′ polyadenylation tail and is produced by the back-splicing of precursor messenger RNAs (mRNAs).^3^ It is highly and stably conserved and has tissue-specific structures that are widely expressed in mammals.^4^ Since the initial identification of circRNA in 1976, many circRNAs have been discovered and identified due to advances in bioinformatics and high-throughput sequencing technologies.^5^,^6^ It has a number of functions, including the regulation of gene expression by sponging microRNAs (miRNAs), protein translation, and interactions with RNA-binding proteins for multiple processes.^7^ CircRNAs are more stable than linear RNAs because of their resistance to RNA exonucleases or RNase R, and this stability in tissues and body fluids makes circRNAs useful as biomarkers for the diagnosis of disease and outcome estimations.^8^,^9^ Regarding ESCC, as well as other cancers, circRNAs with the potential to serve as novel biomarkers have been examined in recent years and their number is increasing.^10^–^16^

Circular FAT1 (circFAT1, circ_0001461) is a non-coding RNA with a cyclic structure that is formed by the back-splicing of exon 2 of the FAT1 gene and head-to-tail binding. Its expression and oncological significance were initially reported for osteosarcoma in 2018^17^ and subsequent studies reported similar findings for other cancer types. CircFAT1 functions as a tumor promoter or suppressor in specific cancers by sponging miRNAs and repressing their downstream pathways;^18^–^22^ however, it has not yet been reported as a prognostic biomarker in any cancers. The present study investigated the biological function of circFAT1 and its potential as a prognostic biomarker in ESCC.

## Materials and Methods

### Patients and Clinical Samples

Fifty-one ESCC patients who underwent radical subtotal esophagectomy with lymphadenectomy at the Kyoto Prefectural University of Medicine Hospital between May 2010 and January 2017 were included in the present study. Thirty-four of 51 patients received neoadjuvant chemotherapy according to the recommendation of the Guidelines for Diagnosis and Treatment of Carcinoma of the Esophagus for stage II and III patients.^23^ The follow-up period for the censored cases was a median of 5.1 years (range 3.6–10.1). Patients with a history of other cancers within 5 years were excluded. Clinicopathological findings and postoperative courses were collected from medical records and databases. Tumor stages were classified based on the 7th edition of the Union for International Cancer Control (UICC)/TNM staging system for esophageal cancer. Formalin-fixed paraffin-embedded (FFPE) slices of tumor tissue and adjacent non-tumorous mucosal tissue were obtained from the resected specimens of ESCC patients.

The present study was approved by the Faculty of Science Ethics Committee at the Kyoto Prefectural University of Medicine (Kyoto, Japan) and was conducted in accordance with the principles of the Declaration of Helsinki. Written informed consent for treatment and research was obtained from all patients.

### Cell Lines

The human ESCC cell lines TE2, TE5, TE8, TE11, TE13, and TE15, and the immortalized fibroblast cell line WI-38, were obtained from the RIKEN BioResource Research Center Cell Bank (Tsukuba, Japan). The human ESCC cell lines KYSE70, KYSE150, and KYSE170 were obtained from the Japanese Collection of Research Bioresources Cell Bank Center (Osaka, Japan). The immortalized human esophageal mucosal cell line Het-1A and immortalized human mesothelial cell line Met-5A were purchased from the American Type Culture Collection (ATCC, Rockville, MD, USA). The human umbilical vein endothelial cell (HUVEC) line was purchased from PromoCell (Heidelberg, Germany). ESCC, WI-38, Het-1A, and Met-5A cell lines were cultured in Roswell Park Memorial Institute (RPMI) medium (Nacalai Tesque, Kyoto, Japan) mixed with 10% fetal bovine serum (FBS; System Biosciences, Palo Alto, CA, USA), 100 µg/mL streptomycin, and 100 U/mL penicillin. HUVEC were cultured in endothelial basal medium (EBM; Lonza, Allendale, NJ, USA) with the endothelial growth supplement SingleQuots (EGM-2; Lonza). All cells were cultured at 37°C in a humidified 5% carbon dioxide incubator.

### RNA Extraction

Total RNA was extracted from FFPE slices using the AllPrep^®^ DNA/RNA FFPE Kit (Qiagen, Hamburg, Germany) and from all cell lines using the miRNeasy^®^ Mini Kit (Qiagen), according to the manufacturer’s instructions. The concentration and quality of all extracted RNA were confirmed using the NanoDrop 1000 spectrophotometer (Thermo Fisher Scientific, Carlsbad, CA, USA).

### Direct Sequencing Analysis

The sequence of the circFAT1 head-to-tail junction was detected by Sanger sequencing. A reverse transcription polymerase chain reaction (RT-PCR) was performed using PrimeSTAR^®^ GXL DNA polymerase (TaKaRa Bio, Kusatsu, Japan) under an annealing temperature of 55 °C, with 10 µg of complementary DNA (cDNA) made from the RNA of ESCC cell lines using the StepOne RT-PCR system (Thermo Fisher Scientific). The accurate lengths of PCR products were detected using agarose gel electrophoresis, and images were captured by the BLook LED transilluminator (BIOHELIX, Keelung City, Taiwan) and a digital camera. PCR products were applied for Sanger sequencing using the BigDye Terminator v1.1 Cycle Sequencing Kit (Thermo Fisher Scientific) and run on an ABI 3500 genetic analyzer (Applied Biosystems, Foster City, CA, USA).

### Quantitative Reverse Transcription Polymerase Chain Reaction (qRT-PCR) Assay for circFAT1 and miR-548g

To investigate the expression of circFAT1, a reverse transcription reaction was performed using 250 ng of RNA extracted from tissues or cell lines with the High Capacity cDNA Reverse Transcription Kit (Applied Biosystems). The linearFAT1 and circFAT1 primers used in the present study were purchased from Integrated Device Technology (IDT, San Jose, CA, USA) (electronic supplementary Table S1). Glyceraldehyde 3-phosphate dehydrogenase (GAPDH; Hs02786991_g1, Applied Biosystems) was used as an internal control for the normalization of gene expression. Quantitative RT-PCR (qRT-PCR) was performed to analyze the expression of circFAT1 in tissues and cell lines using the StepOne Plus PCR system (Applied Biosystems). cDNA was applied to qRT-PCR using Taqpath qPCR Master Mix (ROX; Applied Biosystems) and a specific primer.

In the miR-548g expression analysis, 100 ng of total RNA was applied for the reverse transcription reaction, which was conducted with the TaqMan MicroRNA Reverse Transcription Kit (Applied Biosystems). The primer used in the expression analysis was hsa-miR-548G (002879; Applied Biosystems). RNU6B (001093; Applied Biosystems) was used as the internal control for the normalization of gene expression.

qRT-PCR was performed under the following conditions: at 95 °C for 10 min, 40 cycles at 95 °C for 15 s, and at 60 °C for 1 min. Cycle threshold (*C*_t_) values were calculated using StepOne Software v2.0 (Applied Biosystems), and the results were evaluated using the ΔΔ*C*_t_ method relative to GAPDH and RNU6B.

### RNase R Resistance Assay

Ten microliters of RNase-free solution containing 10 μg of the total RNA of TE2 and TE5 cells was treated with RNase R (Applied Biological Materials, Richmond, BC, Canada). The RNase R mixture was combined with 2 μL of RNase R (20 U), 2 μL of 10× RNase R buffer, and 1 μL of RNase inhibitor (20 U) [Applied Biosystems]. Five microliters of RNase R mixture (test) or RNase-free water (control) was added to 10 µL of RNA solution in a total volume of 20 µL with RNase-free water and incubated at 37°C for 1 h. The products were then immediately placed on ice to stop the reaction and examined using the qRT-PCR assay as described above to measure linear and circFAT1 expression levels.

### Regulation of circFAT1 and miR-548g Expression

Regarding the knockdown of circFAT1 expression, control small-interfering RNA (siRNA) [DS NC-1; IDT] or siRNA targeting circFAT1 (102211666; IDT) was transfected into TE2 and KYSE70 cells on a six-well culture plate at a final concentration of 10 nM using Lipofectamine RNAiMAX (Thermo Fisher Scientific) according to the instructions provided by the manufacturer.

To overexpress miR-548g, a negative control (mirVana miRNA mimic Negative Control #1) or miR-548g mimic (MC13130) was transfected into TE2 and KYSE70 cells with a similar protocol using Lipofectamine RNAiMAX. The downregulation of circFAT1 expression and miR-548g overexpression were confirmed using qRT-PCR.

### Migration and Invasion Assays

We performed migration and invasion assays using the BD BioCoat Matrigel^TM^ Invasion Chamber kit (BD Biosciences, Franklin Lakes, NJ, USA). The upper surface of the 6.4 mm filter with 8 μm pores was coated with Matrigel for the invasion assay but not for the migration assay. Transfected TE2 (1.0 × 10^5^) and KYSE70 cells (3.0 × 10^5^), as described above, were seeded onto the upper Boyden chamber containing RPMI without FBS, and the lower chamber was filled with RPMI with FBS. After cells had been incubated at 37°C for 48 h, migratory or infiltrative cells on the membranes were fixed and stained with the Diff-Quik stain (Sysmex, Kobe, Japan). The nuclei of stained cells were directly counted in six random fields under a microscope, and the mean value was analyzed.

### Statistical Analysis

All statistical analyses were performed using the statistical software JMP^®^ 13.2.1 (2016; SAS Institute Inc. Cary, NC, USA). The Wilcoxon signed-rank test or Student’s *t*-test were used to compare differences between paired or unpaired samples, and categorical variables in two groups were compared using the Chi-square test. Survival curves for recurrence-free survival (RFS) and cancer-specific survival (CSS) were calculated and plotted using the Kaplan–Meier method, and compared using the log-rank test. Cox’s proportional hazard regression model was used to perform a multivariate survival analysis. A *p* value <0.05 was considered to indicate a significant difference. All assays were performed in triplicate.

## Results

### Biological Characteristics of circFAT1 in Esophageal Squamous Cell Carcinoma (ESCC)

**Fig. 1:**
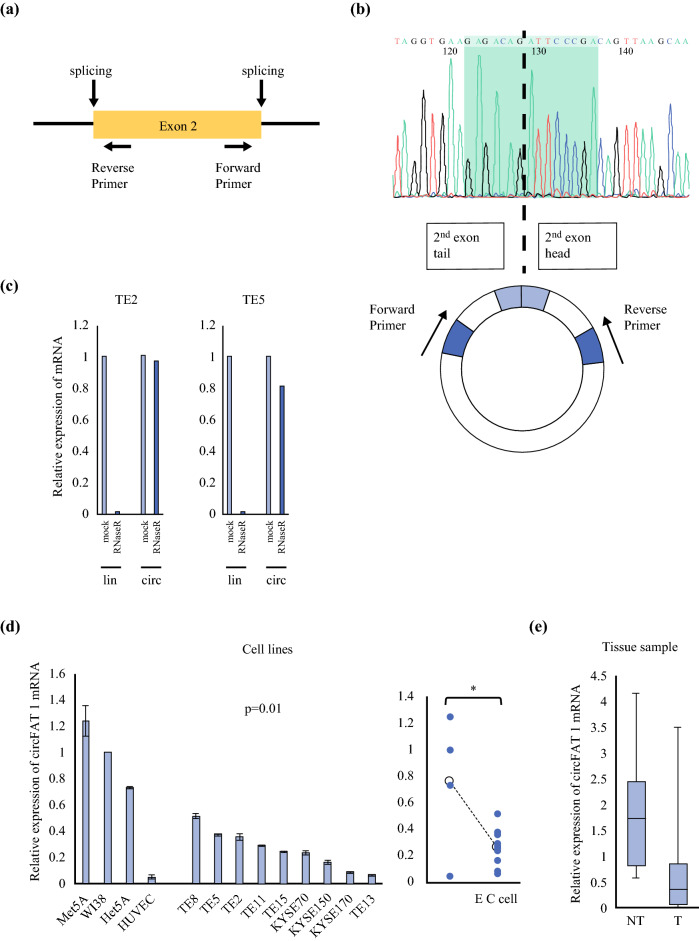
Validation and expression of circFAT1 in ESCC cells and tissues. **a** Schematic figures show the forward and reverse primers designed for exon 2 of the FAT1 gene. **b** Sanger sequencing shows the head-to-tail junction of circFAT1. **c** The expression of linFAT1 and circFAT1 in TE2 and TE5 cells treated with or without RNase R is shown. mRNA levels were evaluated by qRT-PCR and normalized to the mock level. **d** ESCC cell lines (TE2, TE5, TE8, TE11, TE13, TE15, KYSE70, KYSE150, and KYSE170) have lower expression levels of circFAT1 than normal cell lines (Met5A, WI38, Het5A, and HUVEC), as evaluated by qRT-PCR. Data represent the mean ± SD, normalized with GAPDH (*p* = 0.01). **e** Expression of circFAT1 in ESCC tissues extracted from FFPE samples. RNA expression was evaluated by qRT-PCR and analyzed using the Wilcoxon test (*n* = 51; *p* = 0.01). *circFAT1* circular FAT1, *ESCC* esophageal squamous cell carcinoma, *linFAT1* linear FAT1, *mRNA* messenger RNA, *qRT-PCR* quantitative reverse transcription polymerase chain reaction, *SD* standard deviation, *GAPDH* glyceraldehyde 3-phosphate dehydrogenase, *FFPE* formalin-fixed paraffin-embedded

A previous study reported the production of circFAT1 by the back-splicing of the exon 2 part of the FAT1 gene.^17^ Therefore, forward and reverse primers were designed in exon 2 of the FAT1 gene (Fig. 1a). The Sanger sequencing of PCR products confirmed the presence of the sequence containing the circFAT1 head-to-tail junction (Fig. 1b), and showed that the 3′ and 5′ ends of exon 2 formed a head-to-tail junction in ESCC cells. CircFAT1 in ESCC cell lines was more stable than linear FAT1 for the treatment with RNase R (Fig. 1c). The results of qRT-PCR analyses indicated that circFAT1 expression levels were significantly lower in ESCC cell lines than in normal cell lines (*p* = 0.01) (Fig. 1d). CircFAT1 expression levels were significantly lower in ESCC tissue than in the adjacent non-tumorous mucosal tissue of the same patients (*p* = 0.01) (Fig. 1e).

### Effects of circFAT1 Downregulation in ESCC Cell Lines

**Fig. 2:**
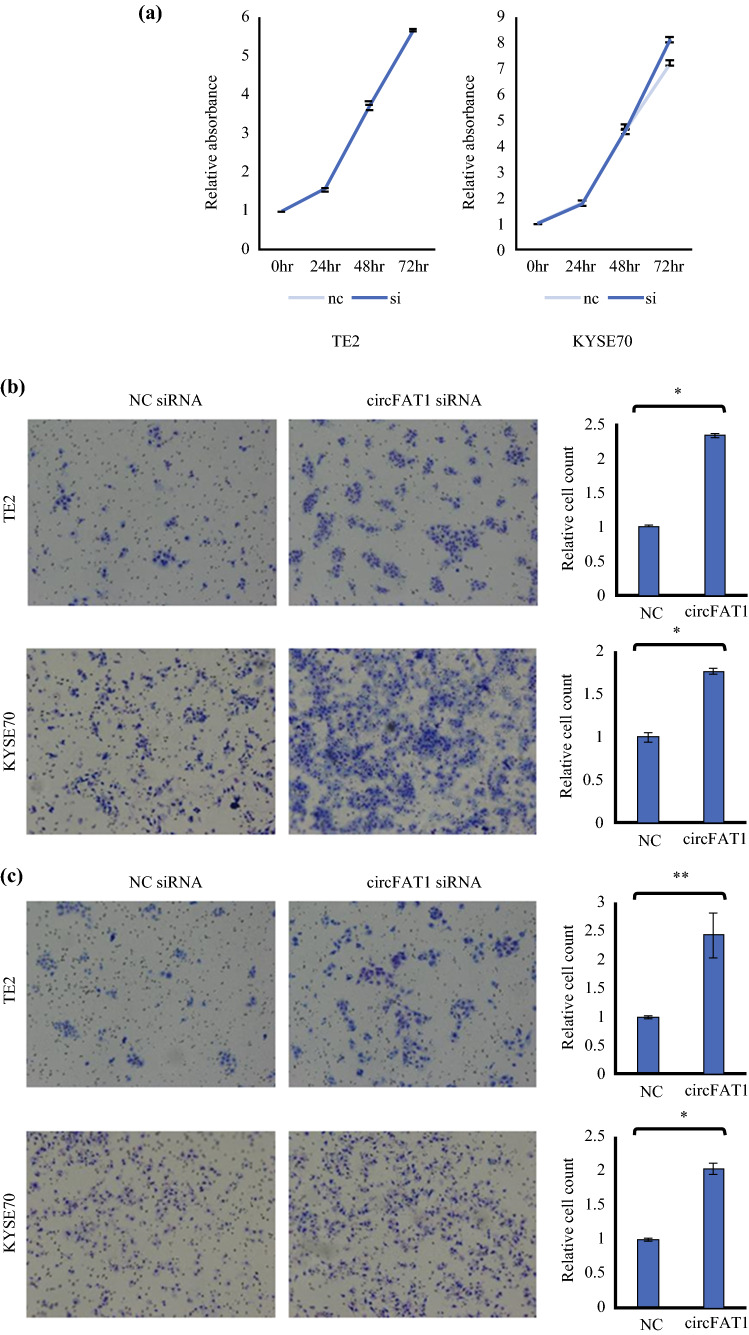
Expression of circFAT1 in ESCC cell lines and the effects of the knockdown of circFAT1 on the migration and invasion abilities in ESCC cells. **a** No significant differences were observed in the proliferation ability of TE2 and KYSE70 cells in which circFAT1 was knocked down. **b** Migration and **c** invasion abilities of TE2 and KYSE70 cells, in which circFAT1 was knocked down by siRNA, were evaluated using the transwell migration assay and Matrigel invasion assay, respectively. Data represent the mean ± SD of the relative cell count. **p < 0.05 and *p < 0.01. *circFAT1* circular FAT1, *ESCC* esophageal squamous cell carcinoma, *siRNA* small-interfering RNA, *SD* standard deviation, *NC* negative control

CircFAT1 expression in TE2 and KYSE70 cells was efficiently downregulated by the transient introduction of siRNA (electronic supplementary Fig. S1). The proliferation of TE2 and KYSE70 cells was not changed by the downregulation of circFAT1 (Fig. 2a); however, the downregulation of circFAT1 resulted in a significant increase in migration and invasion abilities in transwell assays (Fig. 2b, c).

### Effects of miR-548g Expression in ESCC Cell Lines

**Fig. 3:**
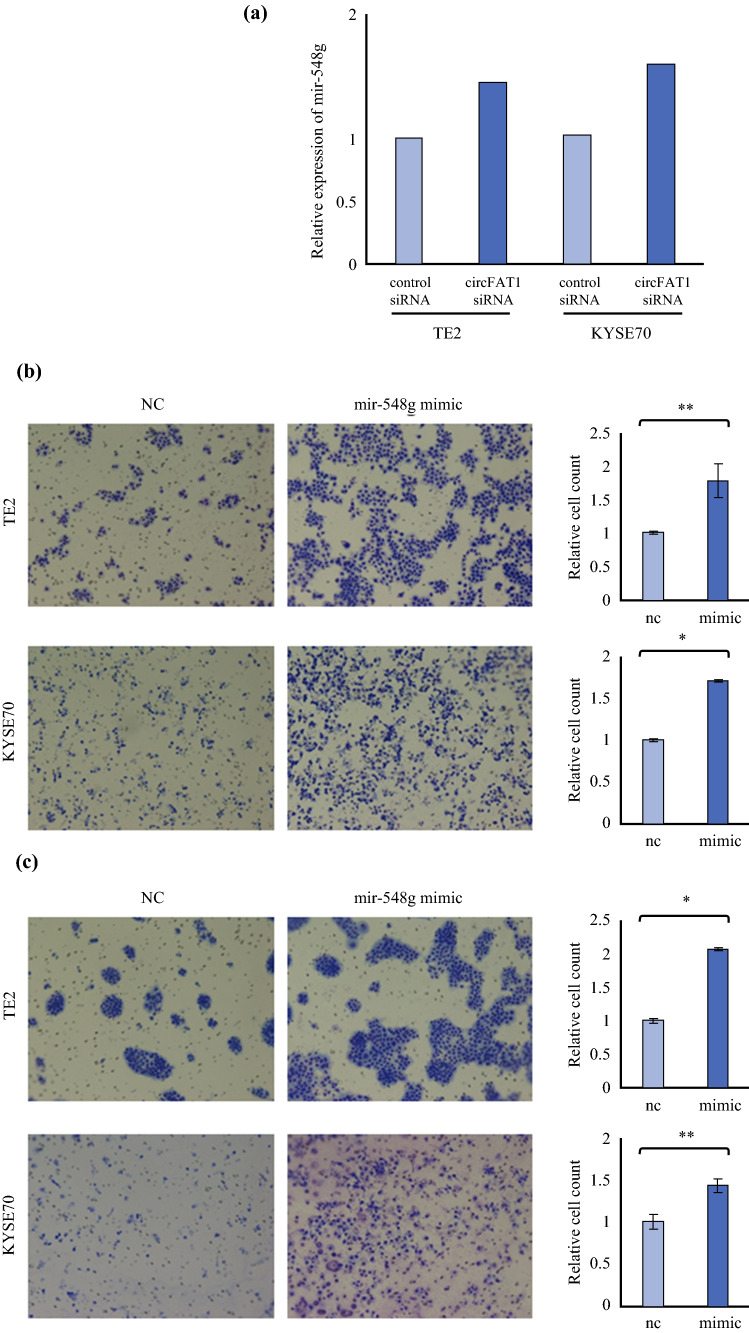
CircFAT1 may sponge miR-548g to suppress tumor progression. **a** Expression of miR-548g in TE2 and KYSE cells in which circFAT1 was knocked down, as evaluated by qRT-PCR. **b** Migration and **c** invasion abilities of miR-548g overexpressing TE2 and KYSE70 cells were evaluated using the transwell migration assay and Matrigel invasion assay, respectively. Data represent the mean ± SD of relative cell counts. ** *p* < 0.05 and * *p* < 0.01. *CircFAT1* Circular FAT1, *qRT-PCR* quantitative reverse transcription polymerase chain reaction, *SD* standard deviation, *NC* negative control

A previous study reported that miR-548g was sponged and downregulated by circFAT1.^18^ The expression of miR-548g was increased by the knockdown of circFAT1 expression based on the results of qRT-PCR (Fig. 3a). miR-548g levels in TE2 and KYSE70 cells were overexpressed by the transient transfection of mimic miR-548g (electronic supplementary Fig. S2). The overexpression of miR-548g promoted migration and invasion abilities, similar to the results of circFAT1 downregulation (Fig. 3b, c).

### Relationships Between circFAT1 Expression and Clinicopathological Factors

CircFAT1 expression in 51 tumor tissues was classified into high- and low-expression groups using the median expression levels of circFAT1 (0.25). High expression levels of circFAT1 in tumor tissue were associated with younger patients (*p* = 0.05), and expression levels were more likely to be higher in patients with no lymph node metastasis (*p* = 0.06) (Table 1). There was no correlation between circFAT1 expression in tumor tissue and pStage (electronic supplementary Fig. S3).

**Table 1:** Significance of tumor tissue circFAT1 expression levels on clinicopathological factors in ESCC patients

Variables	High [*n* = 25]	Low [*n* = 26]	*p* value^b^
Age, years			
< 70	18	12	0.05
≥ 70	7	14	
Sex			
Male	21	19	0.34
Female	4	7	
BMI, kg/m^2^			
< 22	17	16	0.62
≥ 22	8	10	
Location			
Ce/Ut	7	5	0.45
Mt/Lt/Ae	18	21	
Size, mm			
< 45	17	13	0.19
≥ 45	8	13	
Differentiation			
Well–moderate	20	19	0.55
Poor	5	7	
pT factor^a^			
T1–2	9	10	0.85
T3–4	16	16	
pN factor^a^			
N0	13	7	0.06
N1–3	12	19	
pStage^a^			
1–2	15	11	0.20
3–4	10	15	
ly^a^			
0	13	13	0.88
1–3	12	13	
v^a^			
0	11	12	0.87
1–3	14	14	
Absent	11	6	0.11
Present	14	20	
Recurrence			
Local	0	4	0.20
Lymph node	10	12	
Hematogenous	4	4	

*circFAT1* circular FAT1, *ESCC* esophageal squamous cell carcinoma, *BMI* body mass index, *Ce* cervical esophagus, *Ut* upper thoracic, *Mt* middle thoracic, *Lt* lower thoracic, *Ae* abdominal esophagus, *UICC* Union for International Cancer Control, *ly* lymphatic invasion, *v* vascular invasion

^a^ According to the 7th edition of the UICC/TNM staging system

^b^ *p* Values are from the log-rank test

**Fig. 4:**
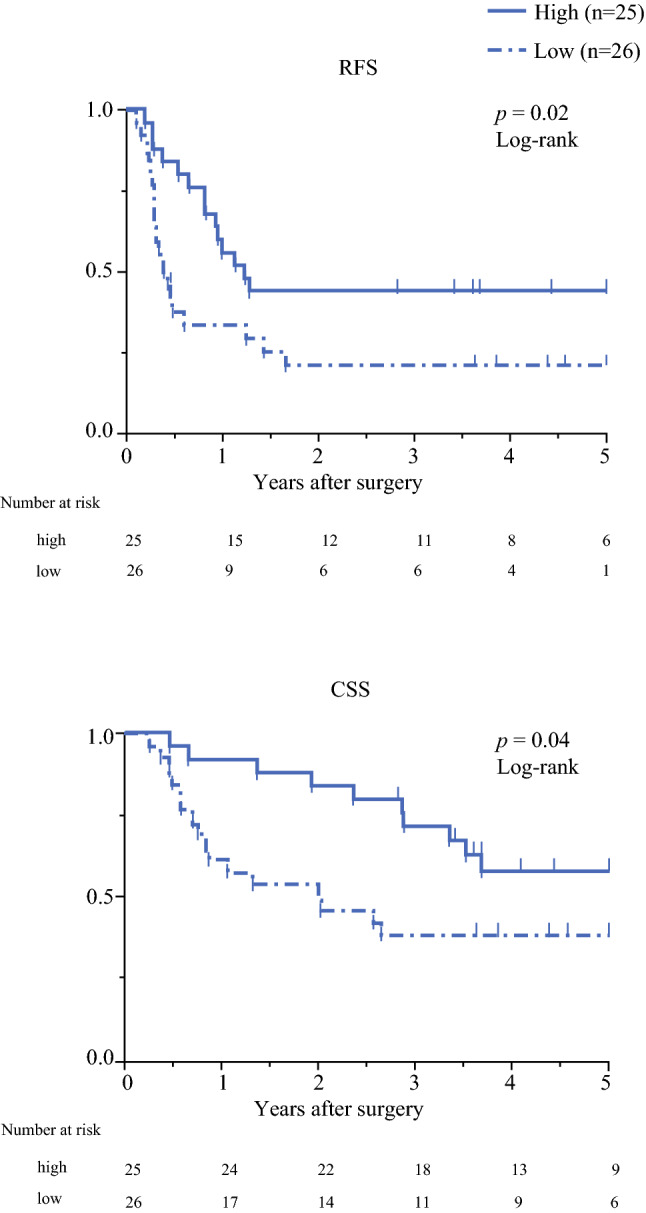
Prognostic impact of circFAT1 expression in ESCC patients. Kaplan–Meier curves for **a** RFS and **b** CSS in ESCC patients according to circFAT1 expression levels in tumor tissue. The log-rank test was used in the statistical analysis. *circFAT1* circular FAT1, *ESCC* esophageal squamous cell carcinoma, *RFS* recurrence-free survival, *CSS* cancer-specific survival

Patients with high expression levels of circFAT1 in tumor tissue showed significantly better 5-year RFS and CSS rates (RFS: 44.0% vs. 20.9%, *p* = 0.02; CSS: 58.0% vs. 38.4%, *p* = 0.04) (Fig. 4). A univariate analysis revealed that pT and pN factors, pStage, and circFAT1 expression levels correlated with RFS and CSS. Furthermore, a multivariate analysis identified low expression levels of circFAT1 and an advanced pStage as independent prognostic factors for RFS (hazard ratio [HR] 2.69, *p* < 0.01; and HR 4.49, *p* < 0.01, respectively) (Table 2) and CSS (HR 2.24, *p* = 0.04; and HR 2.70, *p* = 0.01, respectively) (electronic supplementary Table S2).

**Table 2:** Five-year RFS rate of each clinicopathological factor in ESCC patients and the relationship between the type of recurrence and circFAT1 expression

Variables	*N* = 51	Univariate		Multivariate analysis		
		5-year RFS (%)	*p* value^b^	HR	95% CI	*p* value^b^
Age, years						
≥ 70	21	30.4	0.70			
< 70	30	33.3				
Sex						
Male	40	33.8	0.76			
Female	11	27.2				
BMI, kg/m^2^						
< 22	33	33.3	0.76			
≥ 22	18	30.0				
Location						
Ce/Ut	12	25.0	0.89			
Mt/Lt/Ae	39	34.7				
Size, mm						
< 45	30	42.0	0.09	Ref		
≥ 45	21	19.0		1.02	0.48–2.17	0.95
Differentiation						
Well–moderate	39	37.2	0.06	Ref		
Poor	12	16.7		1.79	0.79–3.79	0.15
pT factor^a^						
T1–2	19	61.7	**< 0.01**	*–*		
T3–4	32	15.6				
pN factor^a^						
N0	20	55.0	**0.01**	*–*		
N1–3	31	17.1				
pStage ^a^						
1–2	26	56.4	**< 0.01**	Ref		
3–4	25	8.0		4.49	2.04–10.50	**< 0.01**
ly^a^						
0	26	36.9	0.46			
1–3	25	25.6				
v^a^						
0	23	41.5	0.39			
1–3	28	28.5				
circFAT1 (expression in T tissue)						
High	25	44.0	**0.02**	Ref		
Low	26	20.9		2.69	1.27–5.92	**< 0.01**

*RFS* recurrence-free survival, *ESCC* esophageal squamous cell carcinoma, *circFAT1* circular FAT1, *RFS* recurrence-free survival, *HR* hazard ratio, *CI* confidence interval, *BMI* body mass index, *Ce* cervical esophagus, *Ut* upper thoracic, *Mt* middle thoracic, *Lt* lower thoracic, *Ae* abdominal esophagus, *UICC* Union for International Cancer Control, *ly* lymphatic invasion, *v* vascular invasion

^a^ According to the 7th edition of the UICC/TNM staging system

^b^ *p* Values are from the log-rank test

## Discussion

miRNA and long non-coding RNA (lncRNA) levels have been examined as potential biomarkers for the early diagnosis of many types of cancers and prediction of outcomes, including ESCC, which has a very poor prognosis.^24^,^25^ CircRNAs have recently emerged as potential biomarkers for the diagnosis of ESCC and outcome estimations.^10^ We herein examined circFAT1, which has not yet been reported in ESCC.

The present results indicated that circFAT1 had a tumor suppressive function and was of greater prognostic value in the high-expression group. High expression levels of circFAT1 were an independent factor for better RFS and CSS. Furthermore, lymph node metastasis was associated with low circFAT1 expression levels. These clinical findings are consistent with the results on migration and invasion abilities in the present study. This is the first study to examine circFAT1 as a prognostic factor in ESCC; however, significant differences were not observed in the form of metastatic recurrence between the high- and low-expression groups due to the number of cases examined (Table 1). CircFAT1 expression levels were significantly higher in younger patients, which suggests that circFAT1 altered immune functions and the expression may be affected by age, as previously reported.^26^

Multidisciplinary treatments, such as chemotherapy, radiation therapy, and surgery for ESCC, are the standard in Japan and are recommended for advanced-stage cancer.^27^ On the other hand, early-stage cancer may be treated by endoscopy or surgical resection alone and has a low incidence of recurrence; however, early recurrence has been reported.^28^ The present results showed early recurrence within 1 year in approximately 70% of patients with low circFAT1 expression levels, and a poor 5-year CCS rate of 38.4% despite radical esophagectomy. Therefore, if tissue circFAT1 expression levels in a biopsy sample or resected specimen are low, additional treatments need to be considered, even for patients with early-stage cancer.

Evidence is accumulating to show that circFAT1 exerts both tumor-promoting^17^,^20^–^22^ and tumor-suppressive effects.^18^,^19^ Tumor-suppressive effects have been reported for adenocarcinomas of the gastrointestinal tract,^18^,^19^ but not for squamous cell carcinoma. The present results showed that circFAT1 expression levels were significantly lower in ESCC tissue than in non-tumorous tissue, which was the case in all cell lines examined. Although the downregulation of circFAT1 did not affect proliferative capacity, it promoted migration and invasive abilities, suggesting the inhibitory effects of circFAT1 on ESCC.

miRNAs are a class of short non-coding RNAs (sncRNAs) that are incorporated into the RNA-induced silencing complex and subsequently hybridize to the 3′-untranslated region of their target mRNAs to repress translation or degrade these mRNAs.^29^ Previous studies reported that miRNAs were involved in various oncological functions, such as cancer cell growth and metastasis.^30^ One function of circRNA is to regulate tumor promotion or suppression by sponging these miRNAs.^9^ CircFAT1 has also been shown to contribute to the repression of its downstream pathway by sponging miRNAs. Its involvement with mir-181b^20^ and mir-375^17^ in osteosarcoma, mir-30a-5p in hepatocellular carcinoma,^22^ mir-873 in papillary thyroid cancer,^21^ and mir-520b in colorectal cancer^19^ has also been reported.

In the present study, we focused on previous findings showing that circFAT1 played a role as a tumor suppressor in gastric cancer by regulating the miR-548g/RUNX1 axis.^18^ miR-548g had five binding sites for circFAT1 and the interaction between miR-548g and circFAT1 was elucidated by a luciferase reporter assay. These findings are also available from the circRNA interactome database developed by the National Institutes of Health, National Institute on Aging (https://circinteractome.nia.nih.gov). In the present study, the downregulation of circFAT1 increased the expression of miR-548g in ESCC cell lines, and the overexpression of miR-548g promoted migration and invasion abilities. Although we could detect miR-548g expression in only 10 clinical samples, there was a slight inverse correlation between circFAT1 and miR-548g expression (electronic supplementary Fig. S4). Therefore, circFAT1 may have a tumor suppressive function in ESCC by sponging miR-548g; however, further research is warranted.

There are several limitations in the present study. Although the expression of circFAT1 in tumor tissues has potential as a prognostic biomarker for ESCC, concrete conclusions were not obtained due to the retrospective nature of the study and the small number of patients examined. Further studies with a larger sample size and prospective design are also needed to show relationships with clinicopathological factors. Although we demonstrated that circFAT1 may alter the prognosis of ESCC by sponging miR-548g, it currently remains unclear whether the circFAT1/miR-548g/RUNX1 axis is also involved in ESCC. Furthermore, the relationships between circFAT1 expression levels and clinicopathological features in clinical samples need to be elucidated in more detail.

## Conclusion

circFAT1 may be a novel prognostic biomarker in ESCC patients. New treatment strategies may be developed in the future based on the tumor suppressive function of circFAT1.

## Supplementary Information

Below is the link to the electronic supplementary material.

Supplementary file1 (DOCX 16 KB)

Supplementary file2 (TIF 52 KB)

Supplementary file3 (TIF 48 KB)

Supplementary file4 (TIF 66 KB)

Supplementary file5 (TIF 52 KB)

Supplementary file6 (DOCX 21 KB)
