# Supplementary material for: Significance of Circular FAT1 as a Prognostic Factor and Tumor Suppressor for Esophageal Squamous Cell Carcinoma
Source: Ann Surg Oncol. 2021 Jun 29;28(13):8508–18. doi: 10.1245/s10434-021-10089-9 (PMC8591040; doi:10.1245/s10434-021-10089-9)
Supplement: Supplementary file 1 — Supplementary file1 (DOCX 16 KB) [file 10434_2021_10089_MOESM1_ESM.docx]

**Supplement figure legends**

**S1. CircFAT1 expression levels after transfection of circFAT1 siRNA, evaluated by qRT-PCR.**

**S2. Effects of mir-548g overexpression on TE2 and KYSE70, evaluated by qRT-PCR.**

**S3. There was no significant correlation between circFAT1 expression in ESCC tumor tissues and pStage factor.**

**S4. Correlation of the expression levels of circFAT1 and mir-548g in ESCC tissues extracted from FFPE samples (n=10). The expression levels were evaluated by qRT-PCR.**
