# Supplementary figures and images for: Significance of Circular FAT1 as a Prognostic Factor and Tumor Suppressor for Esophageal Squamous Cell Carcinoma
Source: Ann Surg Oncol. 2021 Jun 29;28(13):8508–18. doi: 10.1245/s10434-021-10089-9 (PMC8591040; doi:10.1245/s10434-021-10089-9)

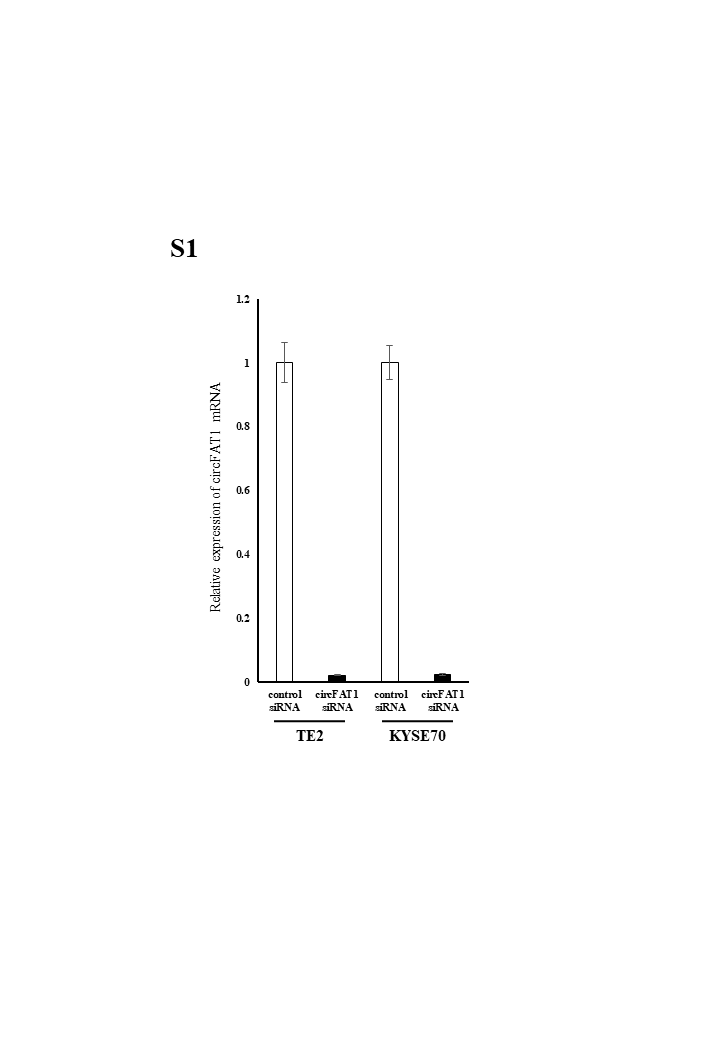

Supplement: Supplementary file 2 — Supplementary file2 (TIF 52 KB) [file 10434_2021_10089_MOESM2_ESM.tif]

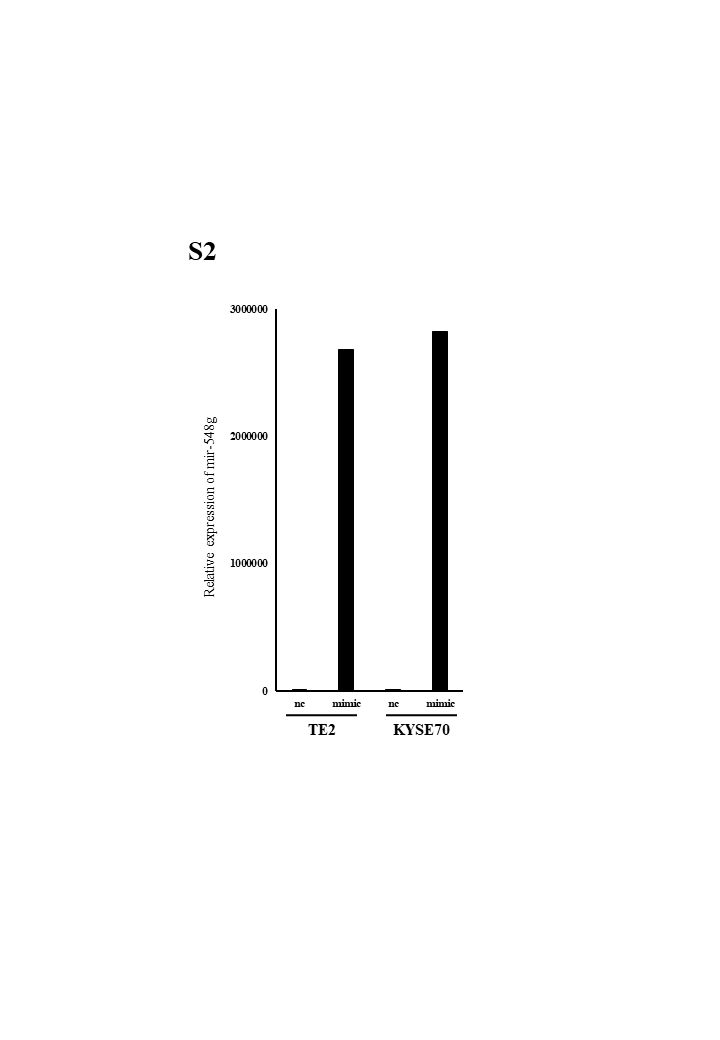

Supplement: Supplementary file 3 — Supplementary file3 (TIF 48 KB) [file 10434_2021_10089_MOESM3_ESM.tif]

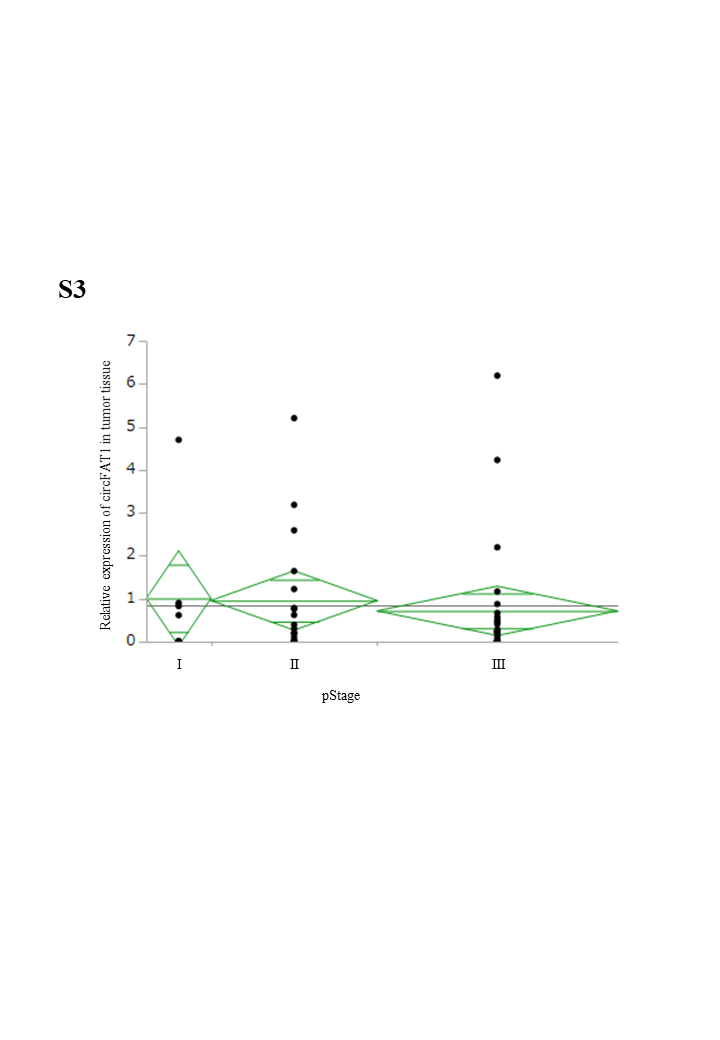

Supplement: Supplementary file 4 — Supplementary file4 (TIF 66 KB) [file 10434_2021_10089_MOESM4_ESM.tif]

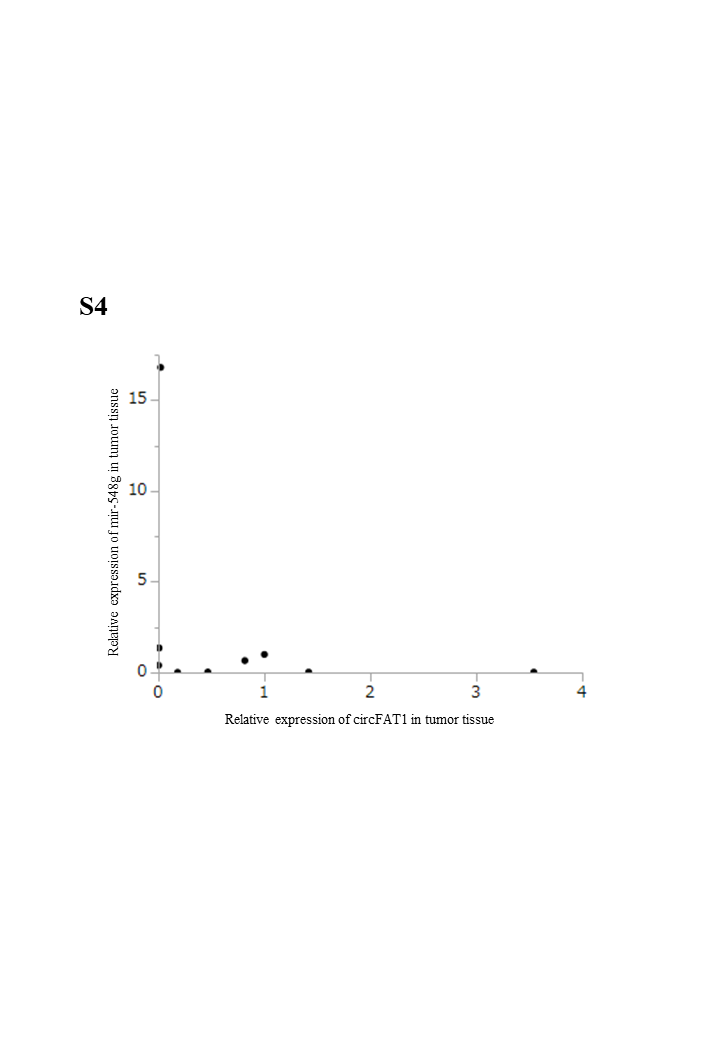

Supplement: Supplementary file 5 — Supplementary file5 (TIF 52 KB) [file 10434_2021_10089_MOESM5_ESM.tif]
