# Supplementary material for: Significance of Circular FAT1 as a Prognostic Factor and Tumor Suppressor for Esophageal Squamous Cell Carcinoma
Source: Ann Surg Oncol. 2021 Jun 29;28(13):8508–18. doi: 10.1245/s10434-021-10089-9 (PMC8591040; doi:10.1245/s10434-021-10089-9)
Supplement: Supplementary file 6 — Supplementary file6 (DOCX 21 KB) [file 10434_2021_10089_MOESM6_ESM.docx]

**Supplement Tables**

**Supplement Table 1. Primers used to evaluate RNA expression in tissues and cell lines by qRT-PCR.**

| **Gene** | **Primer** | |
| --- | --- | --- |
|  | **Forward (5`-3`)** | **Reverse (5`-3`)** |
| **Linear FAT1** | **TCGTAGCCTCCAGGGTAATAG** | **ATGAGGTGATTGATGAGCAGAC** |
| **Circular FAT1** | **AGCAGAAGCAGGAGCAAAG** | **GCTCTGGCGTTGGTGTT** |

**Supplement Table 2. Five-year cancer-specific survival rate of each clinicopathological factor in ESCC patients.**

| **Variables** | | **n=51** | **Univariate** | |  | **Multivariate analysis** | | |
| --- | --- | --- | --- | --- | --- | --- | --- | --- |
|  |  |  | **5-yr CCS**  **(%)** | ***p-*value ^b^** |  | **hazard**  **ratio** | **95% CI** | ***p ^b^*** |
| Age (years) | ≥70  <70 | 21  30 | 36.7  56.3 | *0.29* |  |  |  |  |
| Sex | Male  Female | 40  11 | 49.1  45.4 | *0.91* |  |  |  |  |
| BMI (kg/m^2^) | <22  ≥22 | 33  18 | 43.7  55.5 | *0.42* |  |  |  |  |
| Location | Ce/Ut  Mt/Lt/Ae | 12  39 | 40.0  50.9 | *0.81* |  |  |  |  |
| Size (mm) | <45  ≥45 | 30  21 | 52.3  42.8 | *0.41* |  |  |  |  |
| Differentiation | Well-mod  poor | 39  12 | 56.0  25.0 | *0.06* |  | Ref  2.07 | 0.86 – 4.68 | *0.09* |
| pT factor ^a^ | T1-2  T3-4 | 19  32 | 73.3  33.7 | ***<0.01*** |  | - |  |  |
| pN factor ^a^ | N0  N1-3 | 20  31 | 68.9  34.8 | ***0.02*** |  | - |  |  |
| pStage ^a^ | 1-2  3-4 | 26  25 | 68.5  26.6 | ***<0.01*** |  | Ref  2.70 | 1.18 – 6.73 | ***0.01*** |
| ly ^a^ | 0  1-3 | 26  25 | 61.0  35.5 | *0.13* |  |  |  |  |
| v ^a^ | 0  1-3 | 23  28 | 49.6  46.4 | *0.67* |  |  |  |  |
| circFAT1  (expression in T tissue) | High  Low | 25  26 | 58.0  38.4 | ***0.04*** |  | Ref  2.24 | 1.01 – 5.24 | ***0.04*** |

^a^ According to the 7th edition of the UICC/TNM staging system

^b^ p-values are from the Log-rank test

CSS; cancer-specific survival, BMI; body mass index
